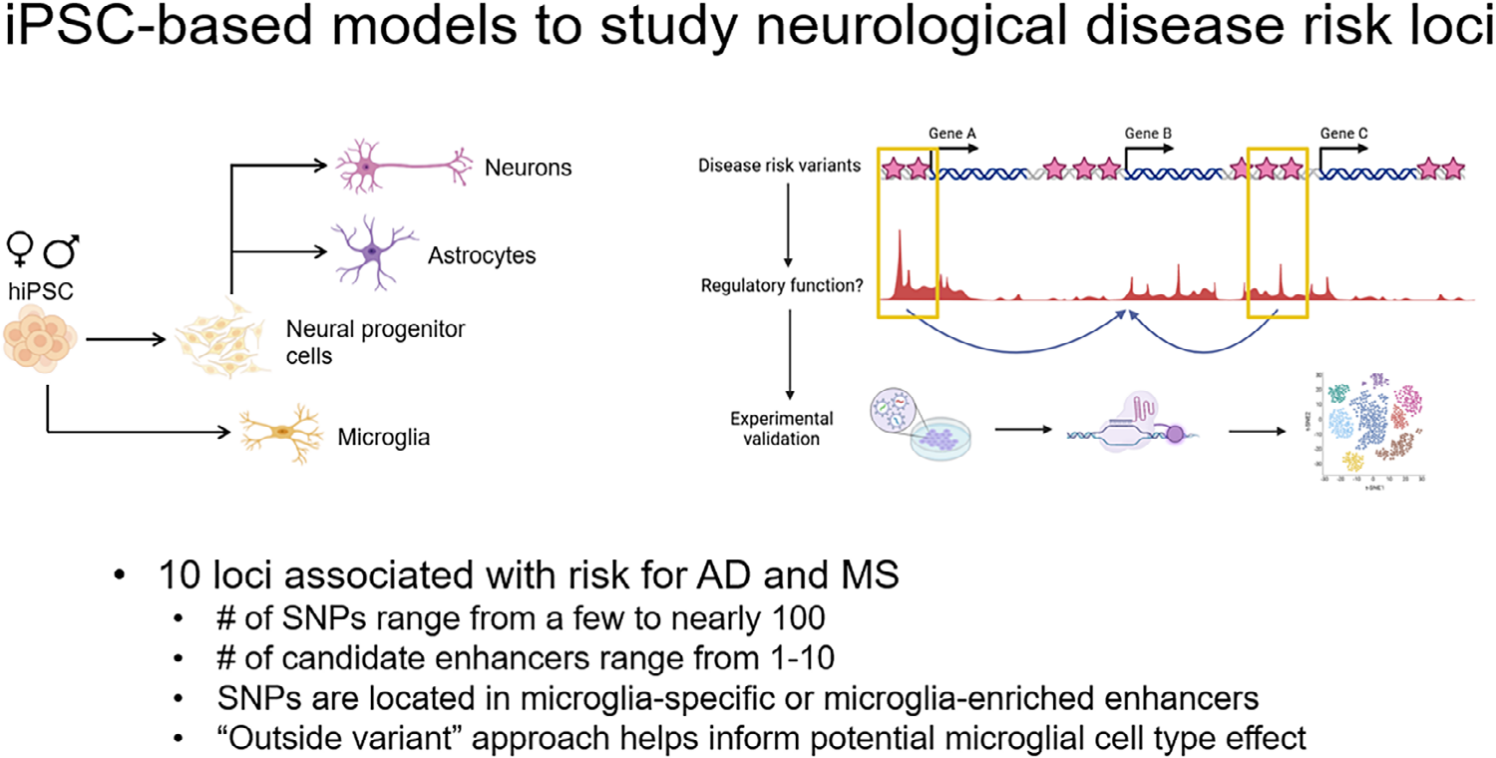# Epigenomics and single cell CRISPR screening to investigate the risk‐modifying role of microglia in Alzheimer’s disease and multiple sclerosis

**DOI:** 10.1002/alz.093591

**Published:** 2025-01-03

**Authors:** Michael D Gallagher, Wenjuan Du, Katheryn EA Hazel, Zeynep Aydin, Yiran Cheng, Bingbing Yuan, George W Bell, Richard A Young, Rudolf Jaenisch, Olivia Corradin

**Affiliations:** ^1^ Whitehead Institute, Cambridge, MA USA; ^2^ Massachusetts Institute of Technology, Cambridge, MA USA

## Abstract

**Background:**

Alzheimer’s disease (AD) and other neurodegenerative diseases (NDs) cause substantial health‐related and economic burdens, but progress towards preventative or ameliorative treatments has been limited. Genome‐wide association studies have identified hundreds of risk loci containing single nucleotide polymorphisms (SNPs) that alter risk for these diseases, but >90% of these SNPs are in noncoding regions, which are cell type‐specific and difficult to study. To address this gap, we have characterized the epigenomes of iPSC‐derived neuronal and glial cells and performed CRISPRi single cell screening to dissect the molecular and cellular mechanisms underlying 10 ND risk loci.

**Method:**

We generated neural progenitors, excitatory neurons, astrocytes and microglia from male and female iPSCs and performed RNA‐seq, H3K27ac ChIP‐seq, and Promoter Capture Hi‐C. We compared these data to those from primary uncultured (ex vivo) human neurons, astrocytes and microglia to compare their enhancer landscapes and ND risk SNP enrichment patterns. To perform CRISPRi single cell screening we integrated a dCas9‐KRAB transgene into an iPSC safe harbor locus and developed a novel lentiviral method that allows for efficient and well‐tolerated delivery of sgRNAs to iPSC‐derived microglia‐like cells (iMGLs).

**Result:**

iMGLs displayed the strongest correlation of their enhancer landscapes with those of their ex vivo counterparts, as well as highly consistent enrichment of AD and multiple sclerosis (MS) risk SNPs in microglia‐specific enhancers. Enhancer/promoter interactions in iMGLs also overlapped significantly with those from ex vivo microglia. As these results suggest that iMGLs are a suitable model for studying ND risk loci, we performed a CRISPRi single cell screen to identify the target genes and pathways affected at 10 AD and MS risk loci.

**Conclusion:**

While the enhancer landscapes of iPSC‐derived neuronal and glial cells vary in similarity to their ex vivo counterparts, they display similar ND risk SNP enrichments in cell type‐specific enhancers, suggesting that disease risk SNP mechanisms are largely recapitulated in iPSC‐derived cells. The iMGL epigenome is notably similar to ex vivo microglia, and CRISPRi screening of AD and MS risk loci in these cells will advance our understanding of these diseases and nominate potential therapeutic targets.